# The outcomes of two different bulking agents (dextranomer hyaluronic acid copolymer and polyacrylate-polyalcohol copolymer) in the treatment of primary vesico-ureteral reflux

**DOI:** 10.1590/S1677-5538.IBJU.2015.0274

**Published:** 2016

**Authors:** Hakan Taşkinlar, Dincer Avlan, Gokhan Berktug Bahadir, Ali Delibaş, Ali Nayci

**Affiliations:** 1 Department of Pediatric Surgery, Mersin University, School of Medicine, Mersin, Turkey;; 2 Department of Pediatric Nephrology, Mersin University, School of Medicine, Mersin, Turkey

**Keywords:** Vesico-Ureteral Reflux, Hyaluronic Acid, Pyran Copolymer, Therapeutics

## Abstract

**Purpose:**

Subureteral injection of bulking agents in the endoscopic treatment of vesicoureteral reflux is widely accepted therapy with high success rates. Although the grade of vesicoureteric reflux and experience of surgeon is the mainstay of this success, the characteristics of augmenting substances may have an effect particularly in the long term. In this retrospective study, we aimed to evaluate the clinical outcomes of the endoscopic treatment of vesicoureteric reflux (VUR) with two different bulking agents: Dextranomer/hyaluronic acid copolymer (Dx/HA) and Polyacrylate polyalcohol copolymer (PPC).

**Materials and Methods:**

A total 80 patients (49 girls and 31 boys) aged 1-12 years (mean age 5.3 years) underwent endoscopic subureteral injection for correction of VUR last six years. The patients were assigned to two groups: subureteral injections of Dx/HA (45 patients and 57 ureters) and PPC (35 patients and 45 ureters). VUR was grade II in 27 ureters, grade III in 35, grade IV in 22 and grade V in 18 ureters.

**Results:**

VUR was resolved in 38 (66.6%) of 57 ureters and this equates to VUR correction in 33 (73.3%) of the 45 patients in Dx/HA group. In PPC group, overall success rate was 88.8% (of 40 in 45 ureters). Thus, Thus, this equates to VUR correction in 31 (88.5%) of the 35 patients.

**Conclusions:**

Our short term data show that two different bulking agent injections provide a high level of reflux resolution and this study revealed that success rate of PPC was significantly higher than Dx/HA with less material.

## INTRODUCTION

The endoscopic injection technique for the treatment of VUR was first described in adults by Matouschek in 1981 and the first clinical series was reported by O’Donnell and Puri in 1984. Although the success rates mainly depend on the VUR grade, surgeon’s experience, anatomic localization of ureters, the nature of the bulking material may also have an effect on the success rates (1-3). Over the past three decades different bulking agents such as collagen, polytetrafluoroethylene (Teflon®), polydimethylsiloxane (Macroplastique®), calcium hydroxyapatite (Coaptite®) have been used (4, 5). Dextranomer/hyaluronic acid copolymer (Dx/HA, Deflux®, Q-Med, Uppsala, Sweden) was introduced into clinical use by Stenberg and Lackgren in 1995. After the approval of Dx/HA in Europe and worldwide clinical studies showing high resolution rates of VUR, it became widely accepted bulking agent in the endoscopic treatment of VUR (6). Dx/HA is composed of dextranomer microspheres and non-animal hyaluronic acid components that forms a viscous gel. Dextranomer microspheres are formed by cross-linking dextran polymers into porous beads of 80-120µm in diameter. A relatively new tissue augmenting material polyacrylate polyalcohol copolymer (PPC, Vantris®, Promedon, Cordoba, Argentina) is a non-biodegradable bulking agent that belongs to the family of acrylics: particles of polyacrylate polyalcohol copolymer. This copolymer is immersed in glycerol with a physiological solution carrier. The average diameter of PPC particles is 300µm with a higher molecular mass. After the implantation of PPC, it induces fibroblastic growth by high superficial electronegativity to be covered by a fibrous capsule. This fibrotic capsule leads to stability and endurance of injected material, affects the local and distant migration and also the success rate (7).

In this retrospective study, we reported the outcomes and success rates of two different bulking agents (Dx/HA and PPC) and analyzed the factors affecting the results in the endoscopic treatment of VUR in children.

## PATIENTS, MATERIALS AND METHODS

We retrospectively reviewed the medical records of all cases that underwent endoscopic treatment for primary grade II-V VUR by the same surgeon between 2007 and 2014. The surgeon had previous experience with different bulking agents such as polytetrafluoroethylene, calcium hydroxyapatite and also with Dx/HA. Patients with anatomic malformations (ureteral duplication, posterior urethral valve, paraureteral diverticula) and neurogenic disorders were excluded from the study. A total of eighty patients (49 females and 31 males) were assigned into two groups: subureteral injection of Dx/HA and subureteral injection of PPC. The children with dysfunctional voiding who were diagnosed by history, uroflowmetry or multichannel urodynamics were primarily allocated to conservative treatment. The radiologic grading of VUR was based on the voiding cystourethrogram (VCUG) according to the international classification system (International Reflux Study Committee) before and after the surgery or during the conservative treatment (8). Dimercaptosuccinic acid (DMSA) renal scan was used to assess renal scarring in preoperative and postoperative follow-up. All patients received antibiotic prophylaxis until VCUG showed spontaneous resolution or definitive cure of the VUR.

**Figure 1:**
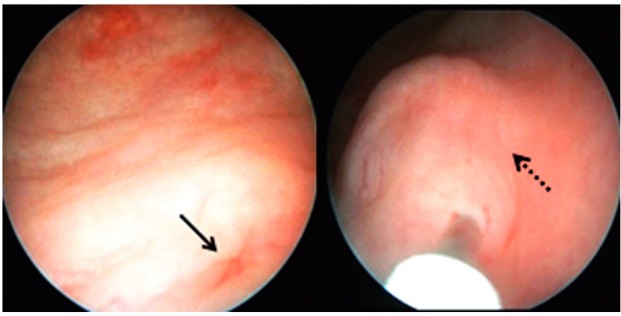
After the injection of bulking material the needle was left in place for 1 minute.

The indications for intervention were breakthrough urinary tract infection (UTI) while on antibiotic prophylaxis, progression of renal scarring and persistent VUR after at least one year of non-operative management. Dx/HA was used as a bulking agent from January 2007 to December 2010, whereas PPC was preferred as injection therapy from January 2011 to December 2014. All injections were performed under general anesthesia. Briefly, the patients were placed in the lithotomy position and the skin was prepared. Bladder was filled about to 70% of the estimated bladder capacity. 9.5Fr pediatric cystoscope with a 5Fr working channel was used for the procedure. The usual technique of subureteral injection is: first the ureteral orifice is determined and the needle is introduced submucosally under the ureteral orifice at 6 o’clock position. After the injection of the bulking material the needle is left in place for 1 minute (Figure-1). Patients were maintained on their antibiotic prophylaxis until the reflux was documented to be absent on postoperative VCUG 3 months after the injection. Patients who failed initial injection were offered a second injection or open surgery and a new VCUG was performed 3 months after the second injection or the surgery. Statistical analysis was performed by using SPSS statistical software, version 11.0 (SPSS Inc. Chicago, III. USA). Means, standard deviations and percentages were used for descriptive statistics. Group comparisons were performed using the independent t test for continuous data and chi-squared test for the categorical data. Values of P<0.05 were considered statistically significant.

## RESULTS

The demographic data and the patient characteristics of both groups are presented in Table-1. There was no significant difference in baseline characteristics between the groups. Dx/HA group included forty-five patients, 29 girls (64%) and 16 boys (36%), with a mean age of 5.3 years (range 1-12 year). The mean follow-up was 32 months (18 to 60 months). In this group, VUR was unilateral in 33 (73%) and bilateral in 12 (27%) patients comprising 57 ureters. VUR was grade II in 15 (26.5%), grade III in 20 (35%), grade IV in 12 (21%) and grade V in 10 (17.5%) ureters. Eighteen patients who had voiding dysfunction were properly treated before intervention.

**Table 1:** Demographic data and patients characteristics of two groups.

	Dx/HA (n=45)	PPC (n=35)
**Mean age (years)**	5.3	5.4
**Gender**		
Male	16 (36%)	15 (43%)
Female	29 (64%)	20 (57%)
**Number of ureter**	57	45
**Laterality**		
Unilateral	33 (73%)	25 (71%)
Bilateral	12 (27%)	10 (29%)
**VUR grade**		
II	15 (26.5%)	12 (27%)
III	20 (35%)	15 (33%)
IV	12 (21%)	10 (22%)
V	10 (17.5%)	8 (18%)

*P* values were all non-significant for the above mentioned data.

Thirty-five patients, 20 girls (57%) and 15 boys (43%), with a mean age of 5.4 years (range 1-11years) and 45 ureters were treated with PPC injection. The mean follow-up was 28 months (10 to 47 months). Twenty-five patients (71%) had unilateral and 10 patients (29%) had bilateral VUR. There were 12 (27 %) ureters graded as II, 15 (33%) as III, 10 (22%) as IV and 8 (18%) as V. Sixteen patients had voiding dysfunction in PPC group.

The comparative success rates between both groups and resolution of VUR in both groups according to the reflux grades are shown in Table-2 and Table-3. VUR was resolved in 30 (52.6 %) of the 57 ureters after a single Dx/HA injection. The success rate rose to 38 (66.6%) in 57 ureters after the second injection and this equates to VUR correction in 33 (73.3%) of the 45 patients. The mean injected volume of Dx/HA was 0.9ml (range 0.4-1.5ml) (Table-2). The residual VUR was observed in 19 ureters (grade V in 9, grade IV in 6 and grade III in 4 ureters). These refluxing ureters were corrected by open surgery.

**Table 2:** Comparison of the success rates after injection treatment with two different bulking agents.

	Dx/HA (ureter=57)	PPC (ureter=45)	P value
**Single injection**	30 (52.6%)	37 (82%)	P<0.05
**Multiple injection**	38 (66.6%)	40 (88.8%)	P<0.05
**Mean injected volume (mL)**	0.9	0.5	P<0.05

**Table 3:** Free of VUR after endoscopic treatment in both groups according to VUR grade.

Dx/HA			PPC		
VUR Grade	RRU (n=57)	Resolved (n=38)		RRU (n=45)	Resolved (n=40)
**II**	15	15 (100%)		12	12 (100%)
**III**	20	16 (80%)		15	15 (100%)
**IV**	12	6 (50%)		10	8 (80%)
**V**	10	1 (10%)		8	5 (62.5%)

In PPC group, the success rate with a single injection was 82% (37 of 45 ureters), while 88.8% (40 of 45 ureters) after the second injection. Thus, this equates to VUR correction in 31 (88.5%) of the 35 patients. The mean injected volume of PPC per ureter was 0.5ml (range 0.2-1.2ml). Residual VUR was observed in 5 ureters (grade V in 3, grade IV in 2 ureters) in this group. All residual VUR were treated with ureteroneocystostomy.

When the results of the injection therapy were compared statistically, a significant difference was found for success rates between the groups. The success rates of PPC injection were significantly higher than the Dx/HA group for both single injection and multiple injections (p<0.05). Furthermore, there was a significant difference for injected volume of bulking agent between both groups. The injected volume of Dx/HA was much more than the volume of PPC (p<0.05). Ureteral obstruction, as a complication, did not emerge after injection or surgical therapy in both groups. Neither adverse reactions nor any signs of toxicity were observed in either group.

After injection, none of cured patients had recurrent UTI (febrile or afebrile) during the follow-up examination. However, nine (5 afebrile, 4 febrile) patients (20%) had UTI after Dx/HA injection, and 4 patients (11.5%) had febrile UTI after PPC injection. All of them showed persistent high grade VUR in both groups.

## DISCUSSION

The concept of the endoscopic correction of VUR offers a minimal invasive treatment in the management of urinary tract infection or renal parenchymal damage associated with reflux. Subureteral injection of bulking agents has recently demonstrated good success rates for endoscopic treatment of VUR and has become increasingly popular for managing VUR. This technique was first described by Matouschek in 1981 and later popularized by Puri and O’Donnell (2). Consequently, many different bulking agents have been used in the endoscopic treatment of VUR until now (4, 5, 9, 10). Since the Food and Drug Administration approved the use of Dx/HA copolymer for endoscopic treatment of VUR, it has gained popularity in many centers in the USA and Europe. It is a biocompatible substance with minimal immunogenic properties and a lack of distant migration (11). Dx/HA is the most studied bulking agent and there is enough long term data existing for understanding its effects. Although, the overall success rates of Dx/HA injection have been reported to be tremendously variable (50-94%) by different authors (12, 13), meta-analysis demonstrated that, on average, 77% of ureters injected with Dx/HA were VUR free 3 months after injection (14, 15). Increasing experience and new injection techniques such as Hydrodistention Implantation Technique (HIT) and double HIT could have led to higher results over time, making this technique also applicable for high- grade VUR (12, 16). On the other hand, many of the studies included in meta-analysis had limited follow-up, only a single VCUG usually within the first 3-6 months postoperatively. Therefore, some recent studies with longer follow-up suggest that these results may not be durable. A recent study by Lee et al. reported a success rate of only 46% in 1 year and studies by Lackgren et al. and Oswald et al. also noted a significant failure rate with extended follow-up (17-19). Moreover, more recent data from the Swedish reflux trial, 20% of previously successfully treated children recurred after 2 years of follow-up, despite relatively high success rates (20). In our study, the overall rate was 66.6% (38/57) ureters after Dx/HA injection and this equates to VUR correction in 73.3% (33/45) of the patients. Although our results appear relatively low, they actually were consistent with other reports.

PPC, the relatively new bulking agent, is a non biodegradable synthetic material which leads to the formation a fibrotic capsule, giving stability and long term permanence. The average diameter of PPC particles is 320µm, thus, it causes a bulkiness that remains stable through time when injected into the soft tissues and reducing the risk of local and distant migration (7, 21). This material was tested with several in-vitro and in-vivo studies. These tests demonstrated that it was non-cytotoxic for cell lines in culture, didn’t cause sensation in mice and no signs of inflammation or necrosis in any organ after implantation (7). On the other hand, the clinical experience with PPC is still very limited. Firstly, in 2010, Ormaecheaet al. reported a multicenter trial from South America (22). In this study, 61 patients with all grades of VUR completed a 1 year follow-up. The number of injected ureters was 88 and the mean injected volume per unit was 0.76ml. The overall success rate of this series was 83.6%. Shortly after this study, Chertin et al. published preliminary data on endoscopic treatment of vesicoureteric reflux with PPC (21). Their series contained thirty-eight children with primary or complex VUR, and the results of the study were quiet satisfactory. The overall success rate was 92.1% in this series. Recently, the results of three years of prospective follow up have been reported by the same group (23). Their success rate was 92.7% after a single injection in all grades of VUR and mean injected volume of PPC per ureter was 0, 7ml (range 0.1, 1ml). Moreover, all patients in this study have had ultrasound scan examination over a 3-year period. The results of ultrasound scan showed the proven evidence of long-term durability of PPC. Another study from Argentina showed 92.3% resolution rate according to the renal refluxing unit and 93.82% according to the number of patients with less than <1ml of material (24). According to our knowledge, there is only one study comparing these two different bulking agents in chronic renal failure adult patients indicating similar effectiveness with Dx/HA and PPC (79% versus %81) (25). In the current study, the overall success rate of PPC injection was 88.8% (40/45 ureters) and the mean injected volume per ureter was 0.5ml. Our results were in concordance with before mentioned studies.

In our study, when demographic data and success rates in both groups were compared, there was not a significant difference in age, gender, laterality and reflux grade between both groups. The overall success rates of PPC were statistically higher than in Dx/HA group. In addition, mean injected volume of Dx/HA was statistically higher than PPC.

One of the most important observations to come out of the literature concerning Dx/HA is the steep learning curve for materials. Kirsch et al. reported a dramatic improvement after the first 20-30 cases in their two surgeon experience, suggesting a 10-15 case learning curve per surgeon (26). Because all injections have been performed in our patients by the same experienced surgeon, the learning curve for Dx/HA injections has not been considered to have negative effects on the current study. PPC is thought as a novel therapy by some authors due to its physiochemical properties, better bulking effect with lower doses and rapid learning curve (24, 27). We also believe that the differences between the results could be based on the biodegradable nature of bulking agents. As mentioned above, the molecular mass of PPC is very high in contrast to Dx/HA. Once injected, the particles of PPC are covered by a fibrotic capsule causing a bulkiness that remains stable through time.

The limitations of our study are that it is a retrospective audit and with short term follow-up. The populations were studied in different periods of time which may affect the learning curve of the surgeon. We also have a longer follow-up period for Dx/HA than PPC that may affect the recurrence rate of each material. However, each material has more than 2-year follow-up period. Another limitation is not having a volume based radiologic study to measure the bulking agent after implantation which may give information about biodegradation or long term stability of these materials.

## CONCLUSIONS

Subureteral injection of bulking agents is a safe, well tolerated, effective minimally invasive outpatient procedure for treatment of children with VUR. Although, our short term data show that two different bulking agent (Dx/HA and PPC) injections provide a high level of reflux resolution, this study suggest that PPC success rates are significantly higher than Dx/HA success rates. However, multicenter studies and/or prospective randomized controlled trials with long term follow-ups are necessary to definitively compare bulking agents in their role as endoscopic therapy for VUR.
